# Knowledge Levels and Training Needs of Disaster Medicine among Health Professionals, Medical Students, and Local Residents in Shanghai, China

**DOI:** 10.1371/journal.pone.0067041

**Published:** 2013-06-24

**Authors:** Tong Su, Xue Han, Fei Chen, Yan Du, Hongwei Zhang, Jianhua Yin, Xiaojie Tan, Wenjun Chang, Yibo Ding, Yifang Han, Guangwen Cao

**Affiliations:** 1 Department of Epidemiology, Second Military Medical University, Shanghai, China; 2 Department of Chronic Diseases, Center for Disease Control and Prevention of Yangpu District, Shanghai, China; Icahn School of Medicine at Mount Sinai, United States of America

## Abstract

**Background:**

Disaster is a serious public health issue. Health professionals and community residents are main players in disaster responses but their knowledge levels of disaster medicine are not readily available. This study aimed to evaluate knowledge levels and training needs of disaster medicine among potential disaster responders and presented a necessity to popularize disaster medicine education.

**Methods:**

A self-reporting questionnaire survey on knowledge level and training needs of disaster medicine was conducted in Shanghai, China, in 2012. A total of randomly selected 547 health professionals, 456 medical students, and 1,526 local residents provided intact information. The total response rate was 93.7%.

**Results:**

Overall, 1.3% of these participants have received systematic disaster medicine training. News media (87.1%) was the most common channel to acquire disaster medicine knowledge. Although health professionals were more knowledgeable than community residents, their knowledge structure of disaster medicine was not intact. Medical teachers were more knowledgeable than medical practitioners and health administrators (*p* = 0.002). Clinicians performed better than public health physicians (*p*<0.001), whereas public health students performed better than clinical medical students (*p*<0.001). In community residents, education background significantly affected the knowledge level on disaster medicine (*p*<0.001). Training needs of disaster medicine were generally high among the surveyed. ‘*Lecture*’ and ‘*practical training*’ were preferred teaching methods. The selected key and interested contents on disaster medicine training were similar between health professionals and medical students, while the priorities chosen by local residents were quite different from health professionals and medical students (*p*<0.001).

**Conclusions:**

Traditional clinical-oriented medical education might lead to a huge gap between the knowledge level on disaster medicine and the current needs of disaster preparedness. Continuing medical education and public education plans on disaster medicine via media should be practice-oriented, and selectively applied to different populations and take the knowledge levels and training needs into consideration.

## Introduction

Over the past decade, the intensity and frequency of natural and man-made disasters have been noticeably increasing all over the world. Hurricane, earthquake, flood, outbreaks of infectious diseases, nuclear leakage, oil spills, and other disasters in recent years have caused huge economic losses, serious environmental disruption and lasting psychological impairment to the survivors [1]–[5]. Community residents are the very ones directly affected by disasters. Therefore, self-rescue and mutual-aid are essential to form the first defense line. Disaster relief and assistance are mainly carried out by medical rescue teams, which are constituted of health professionals from on-call health agencies such as military medical systems and Centers for Disease Control and Prevention (CDC) [6]. Hence, community residents and health professionals as the key components of first responders should be sufficiently trained to perform timely and effective medical rescue [7]. Disaster medicine training, an integrated part of efficient disaster preparedness, is vital for community residents to perform timely self-rescue and mutual-aid and also for health professionals to develop comprehensive skills [8]. Since the ‘9.11′ terrorist attack, many countries have put emphasis on disaster medicine training and have sponsored various researches focusing on a wide range of disaster medicine, including description and assessment of the current disaster medicine training programs in order to improve the efficiency of disaster rescue. However, these researches on disaster medicine have mainly been conducted in developed countries, while data from developing countries are scarce [9]–[14].

From a global perspective, disaster frequently attacks developing countries with weak public health infrastructure and often results in severe consequences. In China, the severe acute respiratory syndrome (SARS) in 2002–2003 resulted in 5,327 cases and 343 deaths [15]. The devastating earthquake in Sichuan, China, in 2008 caused more than 69,000 deaths, 18,341 missing and 374,176 wounded persons [16]. However, disaster medicine has not been included either in the undergraduate curriculum of medical schools or in the continuing medical education in China. In the past decades, Chinese medical education system has experienced flexuous reforms [17]–[19]. Traditional medical education and assessment criteria have been largely clinically oriented, while disaster medicine has been long neglected [20]. Recently, efforts have been made to implement disaster medicine education in China. The current program of disaster medicine education focuses on developing particular small scale training programs, such as short-term training course of disaster nursing for undergraduates, psychosocial training program for mental health workers, and emergency preparedness training program for public health staff [21]–[24]. However, current knowledge status and training needs of main players on disaster medicine were unknown. To the best of our knowledge, only one study surveyed the disaster medicine education needs of health professionals who participated in the earthquake rescue, but their related knowledge was not evaluated [20].

In this study, we evaluated the knowledge levels and training needs in populations that are most likely to be involved in disaster rescue. These data are essential in developing proper medical training programs of disaster medicine.

## Methods

### Participants

Three groups of participants in Shanghai, China, were enrolled in this cross-sectional epidemiological study: health professionals, medical students, and community residents. A stratified cluster random sampling strategy was used to select health professionals and medical students. A total of 600 health professionals were composed of medical practitioners, medical teachers, and health administrators. The medical practitioners were clinicians, public health physicians, nurses, and medical technicians from two comprehensive tertiary hospitals and three CDCs. The medical teachers and 500 medical students were selected from 2 medical schools. Health administrators were from the Municipal Health Bureau and District Health Bureaus. A multi-stage sampling method was used to select 1,600 community residents. We first randomly selected 5 communities in the Yangpu District. In each community, we randomly selected 27, 80, 65, 58, 58 and 32 residents (320 residents per community) at the age of <20 years, 20–30 years, 30–40 years, 40–50 years, 50–60 years and >60 years, respectively, according to the 2010 census data of age composition in Shanghai.

### Questionnaire and Epidemiological Survey

A structured questionnaire for health professionals/medical students was designed by three investigators (TS, HZ, and GC) based on the university examination data bank of emergency medicine, preventive medicine, and health management, as well as published literatures [11], [20]. After two rounds of discussion among the investigators and three rounds of discussion with external experts, final version of the questionnaire was made of three sections. The first section included demographic information such as age, gender, educational level, medical profession, and disaster rescue experience. The second section contained 16 multiple-choice questions (Q1–Q16) as a knowledge test covering various aspects of disaster medicine. In this section, participants could get one score for each correctly answered question and zero for an incorrect answer. The full score was 16. The third section had 5 multiple-choice questions regarding the training needs of disaster medicine. The first questionnaire is presented as Questionnaire S1.

Based on this questionnaire, we designed the second questionnaire for community residents (Questionnaire S2). The second questionnaire had 11 multiple-choice questions (q1–q11) as a knowledge test. Eight questions were included in both questionnaires due to their importance in disaster medicine (Table S1).

Before each survey, trained research assistants would give detailed instructions. The participants were then asked to finish the questionnaire independently.

### Ethics Statement

Informed consent was initially distributed to every candidate study subjects to help them make a fully voluntary decision on participating or declining. Participants who provided their written informed consent were included in this study. The study protocol conformed to the 1975 Declaration of Helsinki and was approved by the ethics committee of Second Military Medical University.

### Statistical Analysis

Descriptive statistics were conducted for demographic characteristics. Differences in categorical variables were determined using the Chi-square test. Analysis of variance (ANOVA) was used to compare the total scores on average among different participants. Student-Newman-Keuls (SNK) test was used to correct for multiple comparisons. Multivariate linear regression was used to analyze the factors contributing independently to the knowledge score. A beta coefficient was calculated to indicate the effect of each independent variable on the score. All tests were two-sided and conducted using SPSS Version 16.0 (SPSS, Chicago, IL). A *p* value of <0.05 was defined as statistically significant.

## Results

### Demographic Characteristics of the Study Participants

A total of 547 (91.2%) health professionals, 456 (91.2%) medical students, and 1,526 (95.4%) community residents provided complete information. Of the 2,529 participants, 1,315 (52.0%) were men and 2,093 (82.8%) were younger than 50 years. Table 1 shows the demographic characteristics. Most of the health professionals had a bachelor’s degree or higher in contrast to community residents (74.6% vs. 23.4%). Health professionals were composed of 380 (69.5%) medical practitioners, 65 (11.9%) medical teachers, and 102 (18.6%) health administrators. Of the 380 medical practitioners, 147 were clinicians, 134 were public health physicians, 77 were nurses and the remaining 22 were medical technicians. The professional titles of health professionals were research assistant (14.0%), senior research assistant (45.3%), assistant professor (33.1%), associate professor (5.1%), and full professor (1.8%). Of medical students, 236 (62.7%) majored in clinical medicine and 170 (37.3%) majored in public health. Among community residents, 52.0% had no stable employment or retired. Of all participants, 197 (7.8%) had disaster relief experience and 33 (1.3%) had ever received systematic training of disaster medicine. For all 2,529 participants, most of them (87.1%) had low or moderate self-estimated knowledge concerning disaster medicine, and media (newspaper, magazine, internet, and TV/radio) was the most common channel to acquire knowledge on disaster medicine.

**Table 1:** Demographic characteristics of study participants (N = 2,529).

	Health professionals		Medical students		Community residents	
Characteristics	n	%	n	%	n	%
**Total**	547	100	456	100	1,526	100
**Gender**						
Male	203	37.1	395	86.6	717	47.0
Female	344	62.9	61	13.4	809	53.0
**Age (years)**						
<30	250	45.7	456	100	544	35.6
30∼50	257	47.0	–	–	586	38.4
>50	40	7.3	–	–	396	26.0
**Educational level**						
Junior college or lower	139	25.4	–	–	1,169	76.6
Bachelor	182	33.3	407	89.3	326	21.4
Master	163	29.8	43	9.4	31	2.0
Doctorate or oversea training	63	11.5	6	1.3	–	–
**Profession**						
Medical practitioner	380	69.5	286*	62.7		
Medical teacher	65	11.9	–	–		
Health administrator	102	18.6	170**	37.3		
Employee	–	–	–	–	446	29.2
civil servant or teacher	–	–	–	–	286	18.7
Others	–	–	–	–	794	52.0
**Disaster relief experience**						
Ever	47	8.6	24	5.3	126	8.3
Never	500	91.4	432	94.7	1,400	91.7
**Systematic training of disaster medicine**						
Ever	25	4.6	9	2.0	–	–
Never	522	95.4	447	98.0	–	–
**Self-estimation of disaster medicine knowledge**						
Well	31	5.7	6	1.3	290	19.0
Moderate	344	62.9	252	55.3	1,069	70.1
Little	172	31.4	198	43.4	167	10.9
**Channels of acquiring information about disaster medicine** #						
Newspaper/magazine/Internet	172	31.4	89	19.5	847	55.5
TV/radio	144	26.3	95	20.8	422	27.7
Lecture/seminar	121	22.1	60	13.2	227	14.9
School education	79	14.4	70	15.4	84	5.5
Communication with others	50	9.1	58	12.7	307	20.1

* Students that major in clinical medicine,

** Students that major in public health management.

# One or more answers were allowed.

### Knowledge Levels

**Figure 1:**
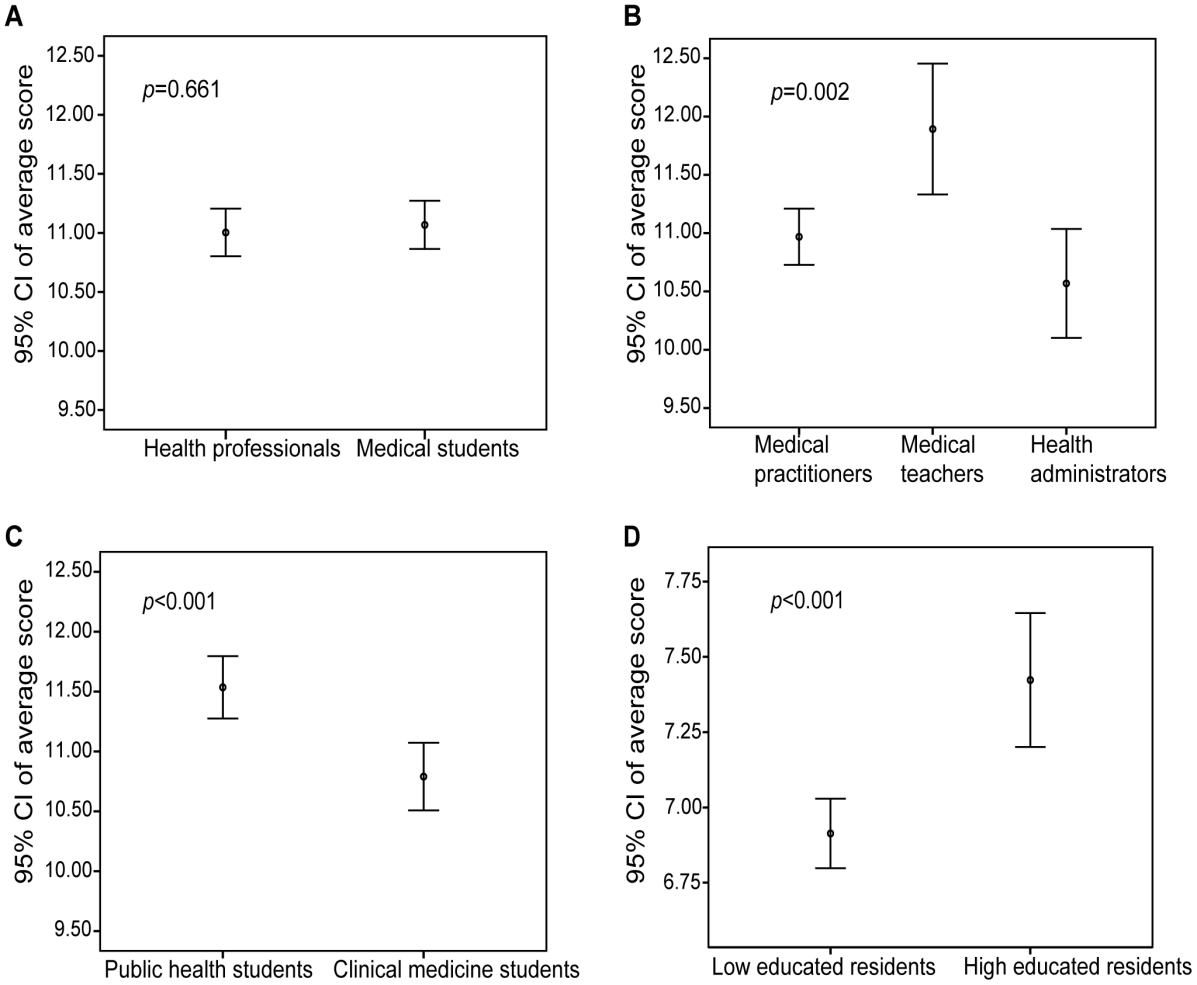
Comparison of the total score on average of disaster medicine knowledge test. A. Health professionals and medical students: no significant difference (*p* = 0.661); B. Three groups of health professionals: total score on average of medical teachers was significantly higher than that of medical practitioners (*p* = 0.010) and health administrators (*p* = 0.001); C. Medical students of two majors: total score on average of public health students was significantly higher than clinical medicine students (*p*<0.001). D. Community residents of different educational levels: total score on average of those with high education background was significantly higher than those without (*p*<0.001).

**Figure 2:**
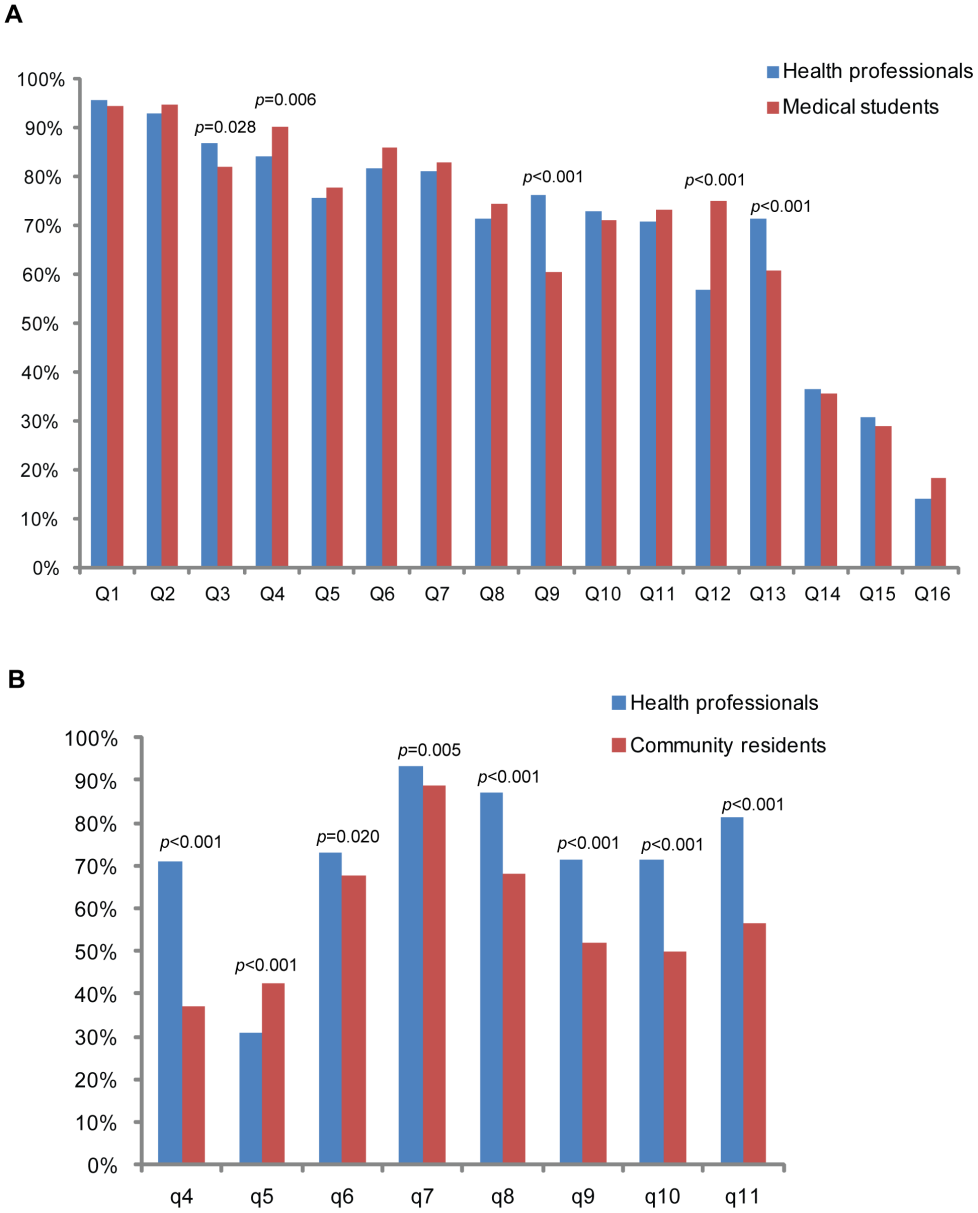
The rates of correctly answering the key questions concerning disaster medicine between different populations. A. Health professionals vs. medical students: *p*<0.05 for Q3 ‘*self-rescue measures in an earthquake*’, Q4 ‘*triage and treatment priority*’, Q9 ‘*concept of first aid ABC*’, Q12 ‘*tourniquet hemostasis*’, and Q13 ‘*skills of psychological assistance in post-disaster relief*’; B. Health professionals vs. community residents: *p*<0.001 for q4 ‘*Cardiopulmonary resuscitation procedure*’, q5 ‘*Difference between remote and urban rescue*’, q8 ‘*Self-rescue measures in an earthquake*’, q9 ‘*Location of temporary toilets during disaster rescue*’, q10 ‘*Skills of psychological assistance in post-disaster relief*’, and q11 ‘*Epidemic prevention strategies after a disaster*’ and *p*<0.05 for q6 ‘*Fracture fixation and transport*’ and q7 ‘*Self-rescue measures in a high-rise fire*’.

Table 2 depicts the correct answer rates to the 16 questions (Q1–Q16) in the knowledge test using the first questionnaire. The questions were correctly answered by >50% of the professionals and students except Q14, Q15, and Q16. Average total score of the knowledge test was 11.00 (95% CI = 10.80–11.21) for health professionals and 11.07 (10.86–11.27) for medical students (*p* = 0.661) (Figure 1A). Although the score of the two populations was not significantly different, there were significant differences in correctly answering individual questions: Q3, Q4, Q9, Q12, and Q13 (Figure 2A).

**Table 2:** Number (%) of health professionals and medical students correctly answering the 16 questions in knowledge test section.

		Health professionals					Medical students			
	Disaster Medicine-related Questions	Total	Medical practitioners	Medical teachers	Health administrators	*p* value	Total	Clinical medicine students	Public health students	*p* value
Q1	Concept of on-site treatment	523 (95.6)	364 (95.8)	64 (98.5)	95 (93.1)	0.250	431 (94.5)	267 (93.4)	164 (96.5)	0.158
Q2	Self-rescue measures in a high-rise fire	509 (93.1)	352 (92.6)	65 (100.0)	92 (90.2)	**0.044**	432 (94.7)	268 (93.7)	164 (96.5)	0.201
Q3	Self-rescue measures in an earthquake	476 (87.0)	334 (87.9)	59 (90.8)	83 (81.4)	0.139	374 (82.0)	222 (77.6)	152 (89.4)	**0.002**
Q4	Triage and treatment priority	461 (84.3)	311 (81.8)	60 (92.3)	90 (88.2)	**0.048**	411 (90.1)	251 (87.8)	160 (94.1)	**0.028**
Q5	Medical evacuation after an earthquake	414 (75.7)	290 (76.3)	60 (92.3)	64 (62.7)	**<0.001**	355 (77.9)	207 (72.4)	148 (87.1)	**<0.001**
Q6	Concept of disaster preparedness	447 (81.7)	303 (79.7)	59 (90.8)	85 (83.3)	0.093	392 (86.0)	237 (82.9)	155 (91.2)	**0.014**
Q7	Epidemic prevention strategies after a disaster	444 (81.2)	305 (80.3)	51 (78.5)	88 (86.3)	0.324	379 (83.1)	227 (79.4)	152 (89.4)	**0.006**
Q8	Location of temporary toilets during disaster rescue	391 (71.5)	262 (68.9)	51 (78.5)	78 (76.5)	0.136	340 (74.6)	203 (71.0)	137 (80.6)	**0.023**
Q9	Concept of first aid ABC (airway, breathing and circulation)	417 (76.2)	301 (79.2)	51 (78.5)	65 (63.7)	**0.004**	276 (60.5)	168 (58.7)	108 (63.5)	0.312
Q10	Fracture fixation and transport	399 (72.9)	278 (73.2)	38 (58.5)	83 (81.4)	**0.005**	324 (71.1)	205 (71.7)	119 (70.0)	0.702
Q11	Cardiopulmonary resuscitation procedure	388 (70.9)	283 (74.5)	49 (75.4)	56 (54.9)	**<0.001**	334 (73.2)	204 (71.3)	130 (76.5)	0.230
Q12	Tourniquet hemostasis	312 (57.0)	208 (54.7)	47 (72.3)	57 (55.9)	**0.029**	343 (75.2)	217 (75.9)	126 (74.1)	0.674
Q13	Skills of psychological assistance in post-disaster relief	391 (71.5)	265 (69.7)	55 (84.6)	71 (69.6)	**0.044**	277 (60.7)	157 (54.9)	120 (70.6)	**0.001**
Q14	Diagnosis of post-traumatic stress disorder (PTSD)	201 (36.7)	141 (37.1)	29 (44.6)	31 (30.4)	0.172	163 (35.7)	104 (36.4)	59 (34.7)	0.721
Q15	Difference between remote area and urban rescue	169 (30.9)	122 (32.1)	18 (27.7)	29 (28.4)	0.650	132 (28.9)	94 (32.9)	38 (22.4)	**0.017**
Q16	Population vulnerability assessment	77 (14.1)	49 (12.9)	17 (26.2)	11 (10.8)	**0.010**	84 (18.4)	55 (19.2)	29 (17.1)	0.563

In health professionals, the score was 10.97 (10.73–11.21), 11.89 (11.33–12.45), and 10.57 (10.10–11.04) for medical practitioners, medical teachers, and health administrators, respectively (*p* = 0.002 for the comparison of three groups) (Figure 1B). For pairwise comparison, SNK test showed that medical teachers’ average score was significantly higher than medical practitioners’ (*p* = 0.010) and health administrators’ (*p* = 0.001), while there was no statistically significant difference between medical practitioners and health administrators (*p*>0.05). The rates of correctly answering 9 questions (Q2, Q4, Q5, Q9, Q10, Q11, Q12, Q13, and Q16) were significantly different among medical practitioners, medical teachers, and health administrators (*p*<0.05) (Table 2). For example, in answering Q5, medical teachers did better than medical practitioners (*p* = 0.004) and health administrators (*p*<0.001). Moreover, the knowledge level was also significantly different among clinicians, public health physicians, nurses, and medical technicians, especially in correctly answering 5 questions (Table S2). Clinicians performed better than public health physicians (*p*<0.001) (Figure S1). In medical students, the score in public health students (11.54, 95% CI = 11.28–11.80) was higher than that in clinical medicine students (10.79, 10.51–11.07) (*p*<0.001) (Figure 1C). The rates of correctly answering 8 questions (Q3, Q4, Q5, Q6, Q7, Q8, Q13, and Q15) were significantly different between the students of 2 majors (*p*<0.05) (Table 2).

Table 3 shows the rate of right responses to the 11 questions (q1–q11) in community residents. The questions were correctly answered by >50% of community residents except q4 and q5. After stratified by educational level, the score of well-educated (bachelor or higher) group (7.42, 7.20–7.65) was significantly higher than that of poor-educated (junior college or lower) group (6.91, 6.80–7.03) (*p*<0.001) (Figure 1D). The rates of correct answers to 7 questions (q2, q4, q5, q7, q8, q9, and q10) were significantly different between the two groups (*p*<0.05).

**Table 3:** Number (%) of community residents correctly answering the 11 questions in knowledge test section.

	Disaster Medicine-related Questions	Total, N = 1,526	High educated residents, N = 357	Low educated residents, N = 1,169	*p* value
q1	Emergency call numbers	1,447 (94.8)	345 (96.6)	1,102 (94.3)	0.077
q2	Position of exit passageway	862 (56.5)	229 (64.1)	633 (54.1)	**0.001**
q3	Self-rescue measures in a nuclear leak	1373 (90.0)	315 (88.2)	1,058 (90.5)	0.211
q4	Cardiopulmonary resuscitation procedure	563 (36.9)	107 (30.0)	456 (39.0)	**0.002**
q5	Difference between remote area and urban rescue	645 (42.3)	185 (51.8)	460 (39.3)	**<0.001**
q6	Fracture fixation and transport	1,031 (67.6)	248 (69.5)	783 (67.0)	0.380
q7	Self-rescue measures in a high-rise fire	1,355 (88.8)	330 (92.4)	1,025 (87.7)	**0.013**
q8	Self-rescue measures in an earthquake	1,038 (68.0)	272 (76.2)	766 (65.5)	**<0.001**
q9	Location of temporary toilets during disaster rescue	792 (51.9)	213 (59.7)	579 (49.5)	**0.001**
q10	Skills of psychological assistance in post-disaster relief	763 (50.0)	203 (56.9)	560 (47.9)	**0.003**
q11	Epidemic prevention strategies after a disaster	863 (56.6)	203 (56.9)	660 (56.5)	0.893

We compared the rates of correctly answering the 8 common questions in both questionnaires (Table S1) between health professionals and community residents. The rates were generally lower in community residents than in health professionals (57.8% *vs*. 72.4%) except q5 (Figure 2B).

Multivariate linear regression analysis indicated that educational level (β = 0.204, *p*<0.001) and professional title (β = 0.142, *p* = 0.008) were significantly associated with an increased knowledge score, whereas age was inversely related to the score (β = −0.193, *p*<0.001), in health professionals. Educational level was the unique factor significantly associated with an increased score in community residents (β = 0.214, *p* = 0.001). Public health major was the factor significantly associated with an increased score in medical students (β = 0.661, *p* = 0.002).

### Training Needs

Table 4 depicts the training needs of health professionals and medical students. The overall opinions on teaching method, course arrangement, and teaching material were consistent among the two groups. More than half of these participants selected ‘*lecture*’, ‘*practical training*’, and ‘*disaster movies or videos*’ as preferred teaching methods. Most participants chose ‘*required course for public health professional*’ as the major training course, and preferred using ‘*national unified textbook*’ as standard teaching material. However, medical teachers considered that ‘*practical training*’ and ‘*disaster movies or videos*’ were not appropriate for teaching disaster medicine, in contrast to medical practitioners and health administrators. Most health administrators believed that disaster medicine training should be a required training subject not only for public health professionals but also for clinicians.

**Table 4:** Training needs of health professionals and medical students (number, %).

	Health professionals					Medical students			
Training Needs	Total	Medicalpractitioners	Medicalteachers	Healthadministrators	*p* value	Total	Clinical medicine students	Public health students	*p* value
**Teaching method**									
Lecture	431 (78.8)	292 (76.8)	54 (83.1)	85 (83.3)	0.242	294 (64.5)	184 (64.3)	110 (64.7)	0.936
Practical training	329 (60.1)	240 (63.2)	25 (38.5)	64 (62.7)	**0.001**	250 (54.8)	139 (48.6)	111 (65.3)	**0.001**
Disaster movies or videos	304 (55.6)	230 (60.5)	21 (32.3)	53 (52.0)	**<0.001**	311 (68.2)	202 (70.6)	109 (64.1)	0.149
Systemic study	257 (47.0)	183 (48.2)	30 (46.2)	44 (43.1)	0.659	174 (38.2)	100 (35.0)	74 (43.5)	0.069
Academic report	229 (41.9)	166 (43.7)	28 (43.1)	35 (34.3)	0.229	110 (24.1)	67 (23.4)	43 (25.3)	0.652
**Course arrangement**									
Required course for public health professional	295 (53.9)	198 (52.1)	36 (55.4)	61 (59.8)	0.371	187 (41.0)	93 (32.5)	94 (55.3)	**0.000**
Selective course for clinician	206 (37.7)	150 (39.5)	20 (30.8)	36 (35.3)	0.352	181 (39.7)	119 (41.6)	62 (36.5)	0.278
Required course for clinician	183 (33.5)	121 (31.8)	15 (23.1)	47 (46.1)	**0.004**	108 (23.7)	67 (23.4)	41 (24.1)	0.867
Selective course for public health professional	160 (29.3)	126 (33.2)	11 (16.9)	23 (22.5)	**0.007**	103 (22.6)	56 (19.6)	47 (27.6)	**0.046**
Informal course	102 (18.6)	71 (18.7)	18 (27.7)	13 (12.7)	0.054	110 (24.1)	75 (26.2)	35 (20.6)	0.174
**Teaching materials**									
National unified textbook	421 (77.0)	292 (76.8)	43 (66.2)	86 (84.3)	**0.025**	209 (45.8)	132 (46.2)	77 (45.3)	0.859
Foreign teaching materials	145 (26.5)	99 (26.1)	19 (29.2)	27 (26.5)	0.866	150 (32.9)	95 (33.2)	55 (32.4)	0.849
Military teaching materials	131 (23.9)	86 (22.6)	21 (32.3)	24 (23.5)	0.239	156 (34.2)	92 (32.2)	64 (37.6)	0.233
Handouts for internal use	73 (13.3)	59 (15.5)	7 (10.8)	7 (6.9)	0.060	100 (21.9)	60 (21.0)	40 (23.5)	0.524
Other	22 (4.0)	14 (3.7)	4 (6.2)	4 (3.9)	0.644	14 (3.1)	8 (2.8)	6 (3.5)	0.661

Table 5 shows disaster medicine training needs of community residents. The majority (88.5%) selected ‘*need to learn disaster medicine*’ and ‘*need of disaster medicine course for children*’. About half of community residents selected ‘*lecture*’ and ‘*practical training*’ as preferred teaching methods. More than 70% of community residents selected ‘*willing to participate in disaster simulation drill regularly*’ and believed that ‘*community volunteer team for disaster relief should be set up and willing to participate volunteer team*’. Compared to community residents with lower educational level, those with higher education background considered that ‘*systemic study*’ was more appropriate for teaching (54.3% *vs*. 43.6%, *p*<0.001).

**Table 5:** Training needs of community residents and their differences between the 2 educational level groups (number, %).

Training Needs	Total, N = 1,526	High-educated, N = 357	Low-educated, N = 1,169	*p* value
**Need to learn disaster medicine**				
Yes	1,350 (88.5)	329 (92.2)	1,021 (87.3)	**0.013**
** **No or does not matter	176 (11.5)	28 (7.8)	148 (12.7)	
**Teaching method**				
Lecture	1,121 (73.5)	286 (80.1)	835 (71.4)	**0.001**
Practical training	775 (50.8)	175 (49.0)	600 (51.3)	0.446
Systemic study	704 (46.1)	194 (54.3)	510 (43.6)	**<0.001**
Disaster movies or videos	661 (43.3)	158 (44.3)	503 (43.0)	0.682
Academic report	152 (10.0)	32 (9.0)	120 (10.3)	0.427
**Need of disaster medicine course for children**				
Yes	1,339 (87.7)	318 (89.1)	1,021 (87.3)	0.211
No	66 (4.3)	18 (5.0)	48 (4.1)	
Does not matter	121 (7.9)	21 (5.9)	100 (8.6)	
**Organize disaster simulation drill in community**				
Willing to participate regularly	1,073 (70.3)	283 (73.7)	810 (69.3)	**0.044**
Willing to participate occasionally	309 (20.2)	74 (20.7)	235 (20.1)	
Not willing to participate	16 (1.4)	3 (0.8)	19 (1.2)	
Does not matter	125 (8.2)	17 (4.8)	108 (9.2)	
**Community volunteer team for disaster relief**				
Should be set up, and willing to participate	1,199 (78.6)	304 (85.2)	895 (76.6)	**0.002**
Should be set up, but not willing to participate	147 (9.6)	23 (6.4)	124 (10.6)	
Should not be set up, and not willing to participate	32 (2.1)	9 (2.5)	23 (2.0)	
Does not matter	148 (9.7)	21 (5.9)	127 (10.9)	

**Figure 3:**
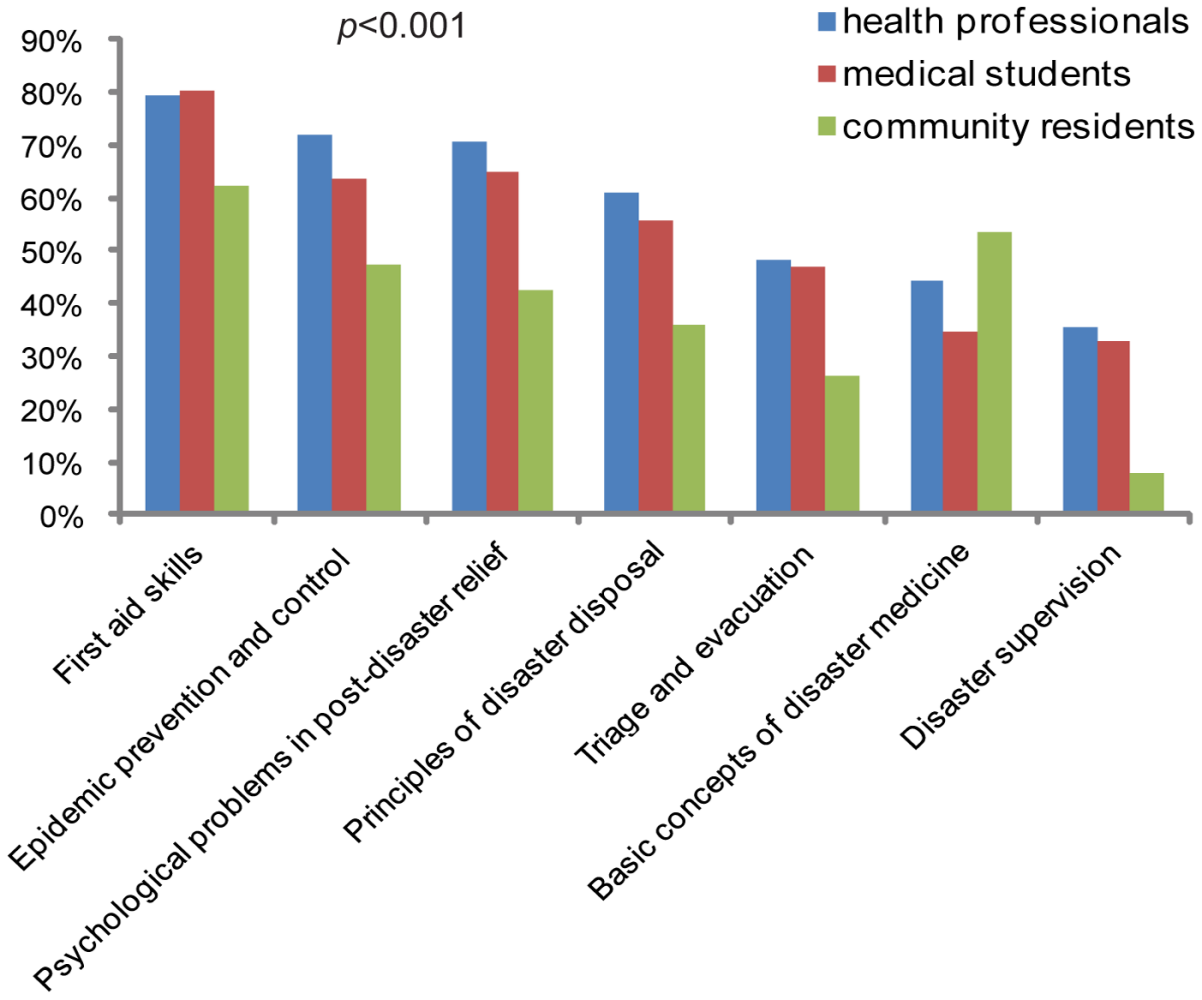
Key contents concerning disaster medicine training prioritized by health professionals, medical students, and community residents.

Figure 3 presents the key contents concerning disaster medicine training prioritized by health professionals, medical students, and community residents. More than 50% of health professionals and medical students selected the contents of ‘*first aid skills*’, ‘*epidemic prevention and control*’, ‘*psychological problems in post-disaster relief*’, and ‘*principles of disaster disposal*’ as important contents; while most community residents chose ‘*first aid skills*’ and ‘*basic concepts of disaster medicine*’ as important contents. Significant differences existed among subgroups within each group of participants. For example, compared to medical practitioners, medical teachers considered that ‘*triage and evacuation*’ was less important (32.2% *vs*. 50.8%, *p* = 0.022) (Table S3).

**Figure 4:**
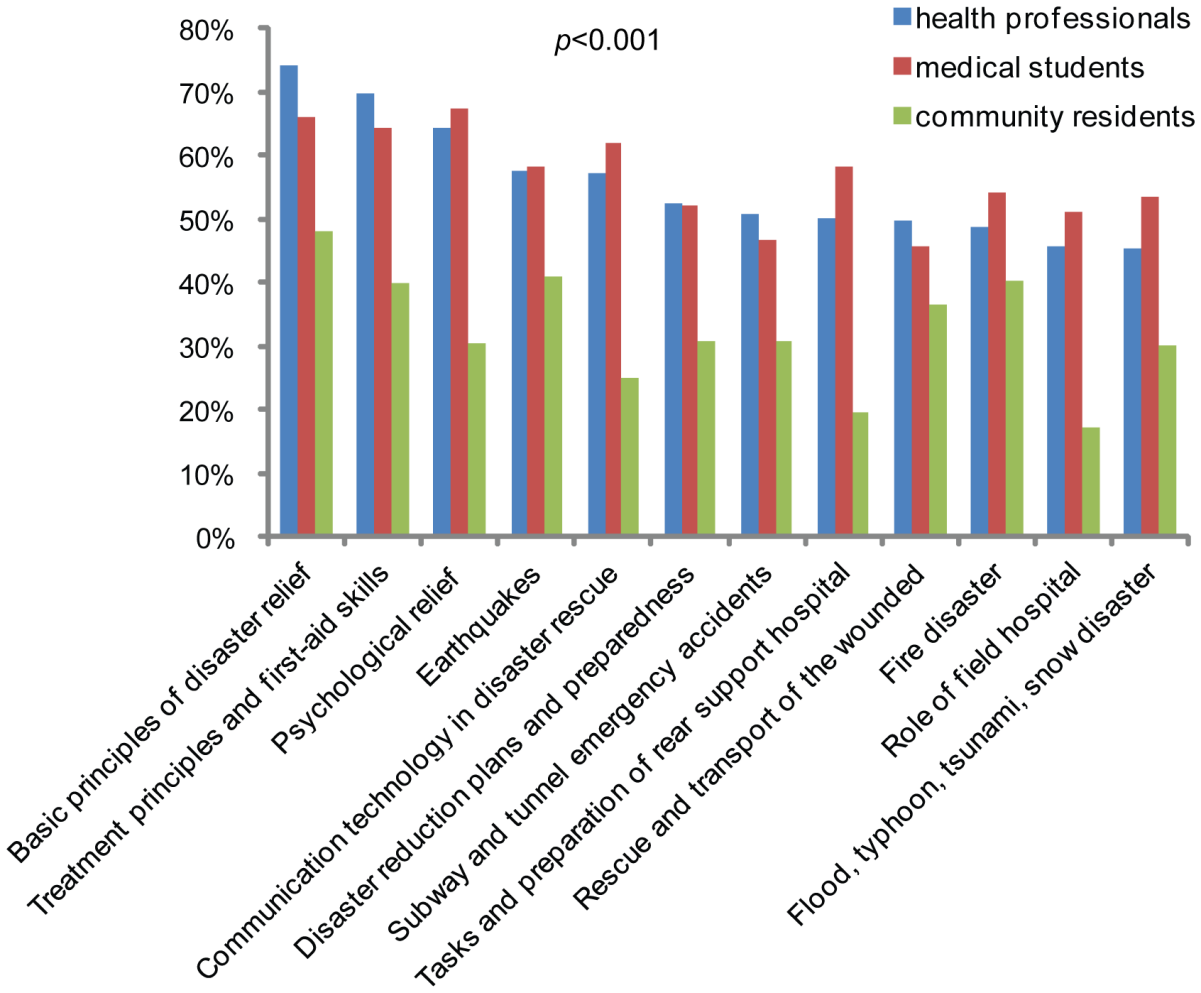
Interested contents concerning disaster medicine training prioritized by health professionals, medical students, and community residents.

Twenty-five items covering most aspects of disaster medicine were provided for the selection of interested training contents (Table S4). Figure 4 presents the most interested contents of disaster medicine training prioritized by health professionals, medical students, and community residents. Health professionals selected ‘*basic principles of disaster rescue*’ (74.0%), ‘*treatment principles and first-aid skills*’ (69.8%), and ‘*psychological relief*’ (64.4%) as the most interested contents, while community residents selected ‘*basic principles of disaster rescue*’ (47.9%) and specific disaster events such as ‘*earthquakes*’ (40.9%) and ‘*fire disaster*’ (40.8%).

## Discussion

In this study, we evaluated the current knowledge levels and training needs of disaster medicine among health professionals, medical students, and community residents in Shanghai, China. In general, our results reflected a high vulnerability of our populations when facing disaster. The knowledge level of disaster medicine was not satisfactory in health professionals except medical teachers. Although the majority of the health professionals received formal medical education, few of them have ever received systematic training of disaster medicine (Table 1). For health professionals and medical students, less accurate responses to Q14, Q15, and Q16 (Table 2) indicate the low levels of knowledge on disaster psychology and disaster administration. The two components have been long neglected and should be added to disaster medicine training and specially addressed to these involved in psychological relief and administrative tasks. Lack of knowledge regarding PTSD is an issue needs to be particularly addressed. Because of the cultural perception in the Chinese society, psychological health hasn’t been widely accepted as a critical component in traditional medical and public health education. Even though there is a rising awareness of its indispensible importance in recent years [1], relevant educational program and public health campaign are still lagging behind. In health professionals, the significant differences among different professions (Figure 1) were mainly presented in their answers to the 9 questions covering 4 aspects: self-help and first-aid skills, triage and evacuation, psychological relief, and population vulnerability assessment. Health administrators did not show their proficiency in disaster administration and disaster rescue organization, for they poorly answered the related questions such as Q5. Leadership training programs could effectively improve the emergency-handling capability of health administrators who might be involved in disaster rescue [25], [26]. Moreover, there were significant differences in knowledge levels among 4 specialties (clinicians, public health physicians, nurses, and medical technicians) of medical practitioners. Clinicians showed higher knowledge level than other specialties, even on the aspect of epidemic prevention and control (Table S2 and Figure S1), which is one of the major tasks of public health physicians. The differences in the knowledge level indicate that the medical education in China had been largely clinically oriented; and little attention has been paid to public health preparedness, especially disaster preparedness. Future training plans should clearly define the roles of public health physicians and health administrators in disaster rescue and enhance their capabilities to meet up-to-date requirements [27]. The main reason of the lack of disaster medicine knowledge for health professionals might be that disaster medicine has rarely been included in medical school curriculum and continuing medical education, and no appropriate public health programs focusing on disaster preparedness. Surprisingly, public health students showed a higher knowledge level than clinical medicine students (*p*<0.001) (Figure 1C). After the SARS outbreak, the importance of public health preparedness has been emphasized with a curriculum restructure for public health major students. In addition to the traditional courses such as epidemiology, training programs for public health preparedness such as health management has been added as the main courses for public health major in some medical schools. However, disaster medicine is being developed as a training course in only a couple of medical schools in China. Our results indicate that future public health physicians are expected to perform better in disaster rescue. Interestingly, the knowledge level of health professionals was inversely related to age, which is in contrast to the general belief that older professionals have more experiences and therefore more knowledgeable. One possible explanation is that the young are more likely to have frequent access to modern media such as the internet and thus gain ‘exposure’ to updated information on disaster medicine.

Community residents displayed very poor knowledge and skills of disaster medicine. Not surprisingly, community residents generally lacked specialty knowledge such as ‘*cardiopulmonary resuscitation procedure*’ and ‘*difference between remote and urban rescue*’ (Table 3 and Figure 2B). An important finding is that community residents with higher education background had higher knowledge level of disaster medicine than those without (Figure 1D). Thus, it is urgent to tailor community training programs for the residents with different education background and popularize disaster medicine education *via* modern media.

This study also pointed out the training needs of disaster medicine. Most participants selected ‘*lecture*’ and ‘*practical training*’ as preferred teaching methods. Most health professionals and medical students suggested that disaster medicine should be a ‘*required course for public health professional*’ and asked for a ‘*national unified textbook*’ as standardized teaching material. Most community residents believed ‘*need to learn disaster medicine*’ and ‘*need of disaster medicine course for children*’, and selected ‘*willing to participate in disaster simulation drill regularly*’ and ‘*community volunteer team for disaster relief should be set up, and willing to participate volunteer team*’ (Table 4, Table 5). These results indicate that the training needs of disaster medicine is very high in Chinese society and disaster medicine trainings should be executed as indispensable courses for health professionals, medical students, and community residents. Meanwhile, the three groups of participants selected some different key and interested contents for disaster medicine training (Figure 3 and Figure 4). This reflects that distinct perception of disaster determines the different needs of disaster medicine training in different populations. Similar differences in several items of the training needs were also presented among the subgroups of study participants. Training programs such as disaster simulation and disaster exercise have proven to be effective and can rapidly deliver core elements of disaster medicine and improve the knowledge level and ability of disaster response [10], [28], [29]. Therefore, future continuing disaster medicine education should focus on developing practice-oriented and core elements-highlighted training courses. Except the high-level interests in ‘*basic principles of disaster relief*’, there were some differences of interested contents among different populations, indicating future training program design should consider both core elements and interests, and customize to different needs. As medical teachers were more knowledgeable in disaster medicine than other populations surveyed (Figure 1B), they should play a leading role in disaster medicine training. Based on these data, we suggest a diagram flow of disaster medicine training as the Shanghai model in Figure S2.

The present survey was conducted in Shanghai, one of the areas with well developed economy and affluent medical resources in China. After further evaluation, the Shanghai model of disaster medicine training suggested in this study should be validated and generalizable to other developing areas where the problem of unmatched economic development and disaster medicine education also exist. These data also provide useful evidence to help developing disaster medicine training plans in other developing world.

The current study had limitations. Our community participants were from one district (Yangpu) in Shanghai chosen by cluster sampling. Sample sizes may influence results if comparing subgroups within clusters. Furthermore, other groups of disaster first responders such as firefighters and military personnel were not included in the current survey. Future studies focusing on these special groups will provide valuable information for disaster preparedness.

In conclusion, this large epidemiological study provided important data concerning knowledge level and training needs among the populations that would be involved in disaster rescue or affected by disasters. From a health education perspective, disaster training programs are urgently needed, with specific emphasis on certain contents, such as psychological relief and administrative skills. Our study enables a more comprehensive evaluation of current disaster preparedness situation and facilitates designing future disaster medicine training programs in China and other developing countries.

## Supporting Information

Figure S1 **Comparisons of the total scores on average and rates of correctly answering 5 important questions among clinicians, public health physicians, nurses, and medical technicians.** A. Comparison of average scores; B. Comparison of correct answer rates.
(TIF)

Figure S2 **Suggested diagram of disaster medicine training (Shanghai model).**
(TIF)

Table S1 List of the same questions in two questionnaires.
(DOC)

Table S2 Comparisons of correctly answering the 16 disaster medicine-related questions among 4 specialties of medical practitioners.
(DOC)

Table S3 Key contents of disaster medicine training prioritized by different study populations.
(DOC)

Table S4 Interested contents of disaster medicine training prioritized by different study populations.
(DOC)

Questionnaire S1 **Questionnaire for health professionals and medical students.**
(DOC)

Questionnaire S2 **Questionnaire for community residents.**
(DOC)
