# Supplementary figures and images for: Knowledge Levels and Training Needs of Disaster Medicine among Health Professionals, Medical Students, and Local Residents in Shanghai, China
Source: PLoS One. 2013 Jun 24;8(6):e67041. doi: 10.1371/journal.pone.0067041 (PMC3691157; doi:10.1371/journal.pone.0067041)

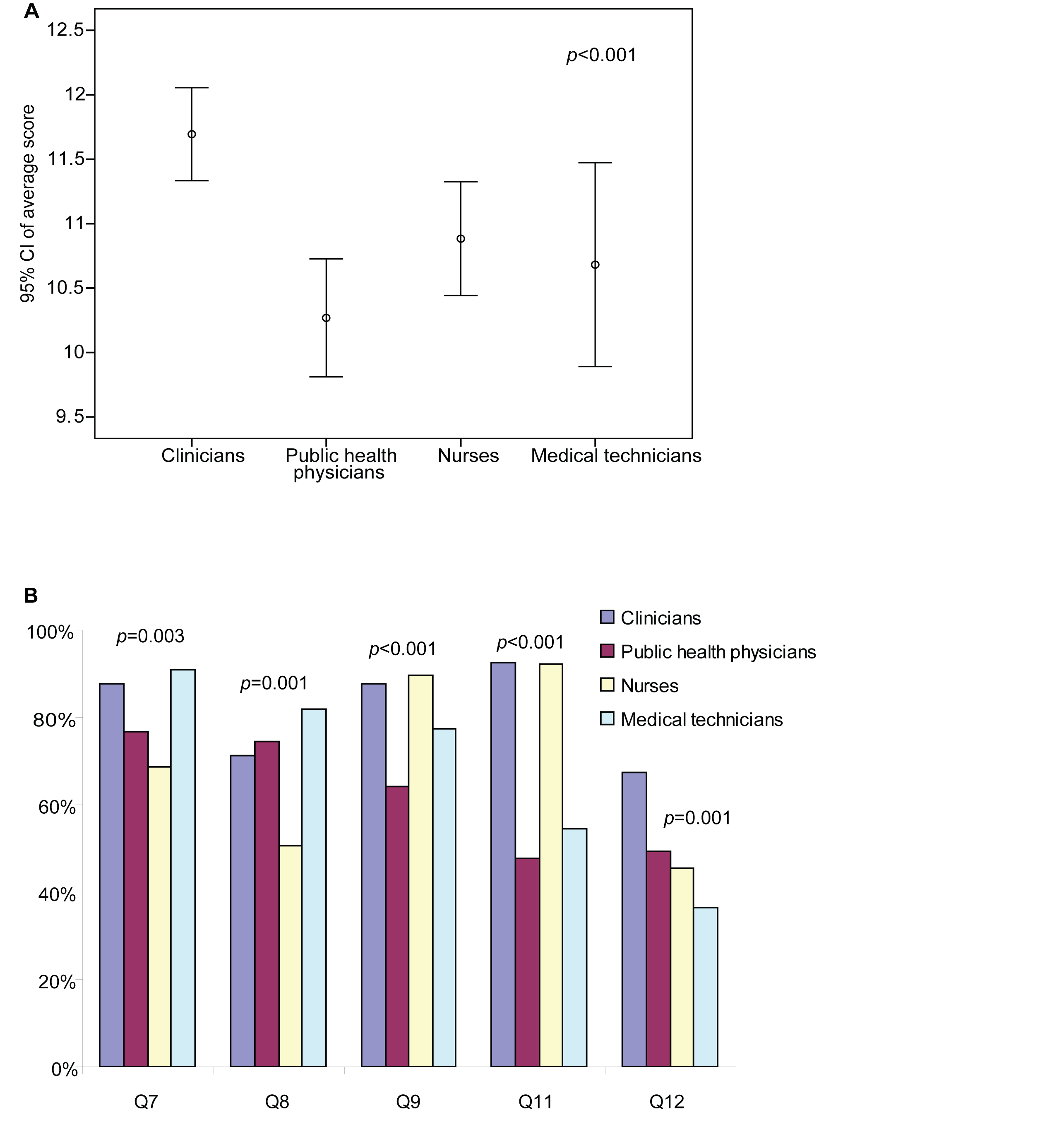

Supplement: Figure S1 — Comparisons of the total scores on average and rates of correctly answering 5 important questions among clinicians, public health physicians, nurses, and medical technicians. A. Comparison of average scores; B. Comparison of correct answer rates. (TIF) [file pone.0067041.s001.tif]

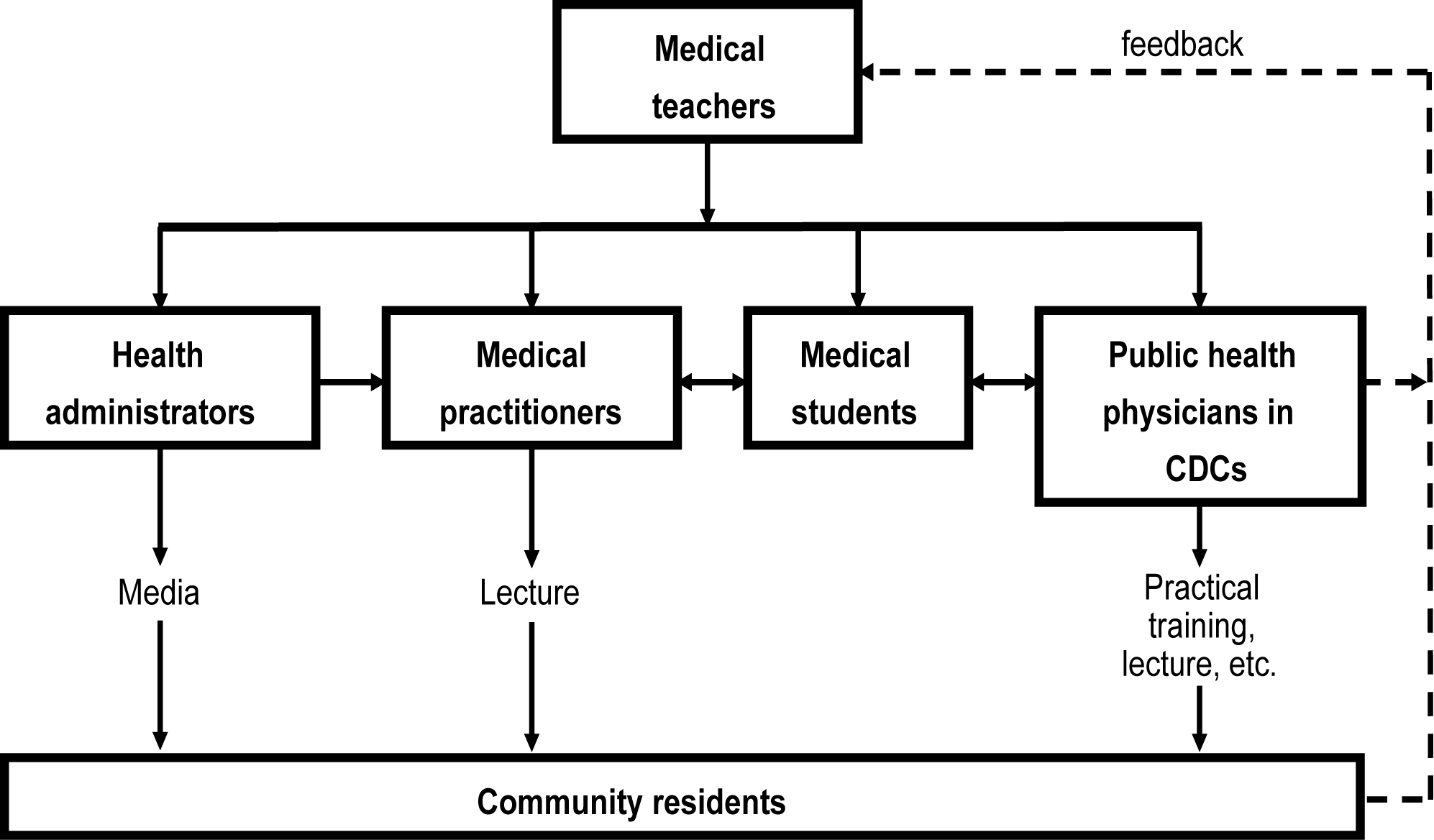

Supplement: Figure S2 — Suggested diagram of disaster medicine training (Shanghai model). (TIF) [file pone.0067041.s002.tif]
