# Supplementary material for: Knowledge Levels and Training Needs of Disaster Medicine among Health Professionals, Medical Students, and Local Residents in Shanghai, China
Source: PLoS One. 2013 Jun 24;8(6):e67041. doi: 10.1371/journal.pone.0067041 (PMC3691157; doi:10.1371/journal.pone.0067041)
Supplement: Table S1 — List of the same questions in two questionnaires. (DOC) [file pone.0067041.s003.doc]

**Table S1.** List of the same questions in two questionnaires.

| **Question No. in questionnaire for health professionals** | **Question No. in questionnaire for community residents** | **Disaster Medicine-related questions** |
| --- | --- | --- |
| Q11 | q4 | Cardiopulmonary resuscitation procedure |
| Q15 | q5 | Difference between remote area and urban rescue |
| Q10 | q6 | Fracture fixation and transport |
| Q2 | q7 | Self-rescue measures in a high-rise fire |
| Q3 | q8 | Self-rescue measures in an earthquake |
| Q8 | q9 | Location of temporary toilets during disaster rescue |
| Q13 | q10 | Skills of psychological assistance in post-disaster relief |
| Q7 | q11 | Epidemic prevention strategies after a disaster |
