# Supplementary material for: Knowledge Levels and Training Needs of Disaster Medicine among Health Professionals, Medical Students, and Local Residents in Shanghai, China
Source: PLoS One. 2013 Jun 24;8(6):e67041. doi: 10.1371/journal.pone.0067041 (PMC3691157; doi:10.1371/journal.pone.0067041)
Supplement: Table S2 — Comparisons of correctly answering the 16 disaster medicine-related questions among 4 specialties of medical practitioners. (DOC) [file pone.0067041.s004.doc]

**Table S2.** Comparisons of correctly answering the 16 disaster medicine-related questions among 4 specialties of medical practitioners.

|  | **Disaster Medicine-related Questions** | **Clinicians, n (%)** | **Public health physicians, n (%)** | **Nurses, n (%)** | **Medical technicians, n (%)** | ***p* value** |
| --- | --- | --- | --- | --- | --- | --- |
| Q1 | Concept of on-site treatment | 140 (95.2) | 129 (96.3) | 74 (96.1) | 21 (95.5) | 0.976 |
| Q2 | Self- rescue measures in a high-rise fire | 139 (94.6) | 120 (89.6) | 72 (93.5) | 21 (95.5) | 0.391 |
| Q3 | Self- rescue measures in an earthquake | 132 (89.8) | 118 (88.1) | 63 (81.8) | 21 (95.5) | 0.225 |
| Q4 | Triage and treatment priority | 124 (84.4) | 107 (79.9) | 63 (81.8) | 17 (77.3) | 0.731 |
| Q5 | Medical evacuation after an earthquake | 117 (79.6) | 94 (70.1) | 59 (76.6) | 20 (90.9) | 0.098 |
| Q6 | Concept of disaster preparedness | 122 (83.0) | 102 (76.1) | 62 (80.5) | 17 (77.3) | 0.539 |
| Q7 | Epidemic prevention strategies after a disaster | 129 (87.8) | 103 (76.9) | 53 (68.8) | 20 (90.9) | **0.003** |
| Q8 | Location of temporary toilets during disaster rescue | 105 (71.4) | 100 (74.6) | 39 (50.6) | 18 (81.8) | **0.001** |
| Q9 | Concept of first aid ABC (airway, breathing and circulation) | 129 (87.8) | 86 (64.2) | 69 (89.6) | 17 (77.3) | **<0.001** |
| Q10 | Fracture fixation and transport | 110 (74.8) | 96 (71.6) | 56 (72.7) | 16 (72.7) | 0.945 |
| Q11 | Cardiopulmonary resuscitation procedure | 136 (92.5) | 64 (47.8) | 71 (92.2) | 12 (54.5) | **<0.001** |
| Q12 | Tourniquet hemostasis | 99 (67.3) | 66 (49.3) | 35 (45.5) | 8 (36.4) | **0.001** |
| Q13 | Skills of psychological assistance in post-disaster relief | 111 (75.5) | 84 (62.7) | 54 (70.1) | 16 (72.7) | 0.134 |
| Q14 | Diagnosis of post-traumatic stress disorder (PTSD) | 54 (36.7) | 47 (35.1) | 32 (41.6) | 8 (36.4) | 0.824 |
| Q15 | Difference between remote area and urban rescue | 46 (31.3) | 44 (32.8) | 29 (37.7) | 3 (13.6) | 0.203 |
| Q16 | Population vulnerability assessment | 26 (17.7) | 16 (11.9) | 7 (9.1) | 0 (0.0) | 0.061 |
