# Supplementary material for: Knowledge Levels and Training Needs of Disaster Medicine among Health Professionals, Medical Students, and Local Residents in Shanghai, China
Source: PLoS One. 2013 Jun 24;8(6):e67041. doi: 10.1371/journal.pone.0067041 (PMC3691157; doi:10.1371/journal.pone.0067041)
Supplement: Table S3 — Key contents of disaster medicine training prioritized by different study populations. (DOC) [file pone.0067041.s005.doc]

|  | **Health professionals** | | | | | **Medical students** | | | | **community residents** | | | |
| --- | --- | --- | --- | --- | --- | --- | --- | --- | --- | --- | --- | --- | --- |
| **Key content** | **Total** | **Medical**  **practitioners** | **Medical teachers** | **Health administrators** | ***p* value** | **Total** | **Clinical medicine students** | **Public health students** | ***p* value** | **Total** | **High educated residents** | **Low educated residents** | ***p* value** |
| First aid skills | 433 (79.2) | 316 (83.2) | 47 (72.3) | 70 (68.6) | **0.002** | 366 (80.3) | 226 (79.0) | 140 (82.4) | 0.387 | 948 (62.1) | 241 (67.5) | 707 (60.5) | **0.017** |
| Epidemic prevention and control after a disaster | 394 (72.0) | 277 (72.9) | 45 (69.2) | 72 (70.6) | 0.779 | 290 (63.6) | 171 (59.8) | 119 (70.0) | **0.028** | 721 (47.2) | 172 (48.2) | 549 (47.0) | 0.687 |
| Psychological problems in post-disaster relief | 385 (70.4) | 278 (73.2) | 39 (60.0) | 68 (66.7) | 0.066 | 295 (64.7) | 182 (63.6) | 113 (66.5) | 0.540 | 646 (42.3) | 168 (47.1) | 478 (40.9) | **0.039** |
| The principles of disaster disposal | 333 (60.9) | 232 (61.1) | 40 (61.5) | 61 (59.8) | 0.967 | 254 (55.7) | 152 (53.1) | 102 (60.0) | 0.154 | 546 (35.8) | 160 (44.8) | 386 (33.0) | **<0.001** |
| Triage and evacuation | 264 (48.3) | 193 (50.8) | 21 (32.3) | 50 (49.0) | **0.022** | 213 (46.7) | 131 (45.8) | 82 (48.2) | 0.615 | 401 (26.3) | 120 (33.6) | 281 (24.0) | **<0.001** |
| Basic concepts of disaster medicine | 242 (44.2) | 173 (45.5) | 26 (40.0) | 43 (42.2) | 0.635 | 157 (34.4) | 93 (32.5) | 64 (37.6) | 0.265 | 818 (53.6) | 190 (53.2) | 628 (53.7) | 0.868 |
| Disaster supervision | 194 (35.5) | 125 (32.9) | 29 (44.6) | 40 (39.2) | 0.129 | 149 (32.7) | 83 (29.0) | 66 (38.8) | **0.031** | 122 (8.0) | 18 (5.0) | 104 (8.9) | **0.019** |

**Table S3.** Key contents of disaster medicine training prioritized by different study populations.
