# Supplementary material for: Knowledge Levels and Training Needs of Disaster Medicine among Health Professionals, Medical Students, and Local Residents in Shanghai, China
Source: PLoS One. 2013 Jun 24;8(6):e67041. doi: 10.1371/journal.pone.0067041 (PMC3691157; doi:10.1371/journal.pone.0067041)
Supplement: Table S4 — Interested contents of disaster medicine training prioritized by different study populations. (DOC) [file pone.0067041.s006.doc]

**Table S4.** Interested contents of disaster medicine training prioritized by different study populations.

|  | **Health professionals** | | | | | **Medical students** | | | | **Community residents** | | | |
| --- | --- | --- | --- | --- | --- | --- | --- | --- | --- | --- | --- | --- | --- |
| **Interested content** | **Total** | **Medical practitioners** | **Medical teachers** | **Health administrators** | ***p* value** | **Total** | **Clinical medicine students** | **Public health students** | ***p* value** | **Total** | **High educated residents** | **Low educated residents** | ***p* value** |
| Basic principles of disaster relief | 405 (74.0) | 282 (74.2) | 44 (67.7) | 79 (77.5) | 0.370 | 301 (66.0) | 191 (66.8) | 110 (64.7) | 0.651 | 731 (47.9) | 172 (48.2) | 559 (47.8) | 0.905 |
| Treatment principles and first-aid skills | 382 (69.8) | 267 (70.3) | 47 (72.3) | 68 (66.7) | 0.702 | 293 (64.3) | 176 (61.5) | 117 (68.8) | 0.117 | 608 (39.8) | 177 (49.6) | 431 (36.9) | **<0.001** |
| Psychological relief | 352 (64.4) | 248 (65.3) | 41 (63.1) | 63 (61.8) | 0.786 | 308 (67.5) | 192 (67.1) | 116 (68.2) | 0.808 | 466 (30.5) | 119 (33.3) | 347 (29.7) | 0.190 |
| Earthquakes | 315 (57.6) | 225 (59.2) | 35 (53.8) | 55 (53.9) | 0.511 | 265 (58.1) | 168 (58.7) | 97 (57.1) | 0.725 | 622 (40.9) | 155 (43.4) | 467 (39.9) | 0.243 |
| Importance of modern communication technology in disaster rescue | 312 (57.0) | 223 (58.7) | 26 (40.0) | 63 (61.8) | **0.011** | 282 (61.8) | 181 (63.3) | 101 (59.4) | 0.410 | 379 (24.8) | 111 (31.1) | 268 (22.9) | **0.002** |
| National and local disaster reduction plans and preparedness against disaster | 286 (52.3) | 204 (53.7) | 34 (52.3) | 48 (47.1) | 0.493 | 238 (52.2) | 156 (54.5) | 82 (48.2) | 0.192 | 468 (30.7) | 131 (36.7) | 337 (28.8) | **0.005** |
| Subway and tunnel emergency accidents | 278 (50.8) | 198 (52.1) | 33 (50.8) | 47 (46.1) | 0.557 | 213 (46.7) | 122 (42.7) | 91 (53.5) | **0.024** | 470 (30.8) | 107 (30.0) | 363 (31.1) | 0.699 |
| Tasks and preparation of rear support hospital in disaster rescue | 273 (49.9) | 211 (55.5) | 17 (26.2) | 45 (44.1) | **<0.001** | 266 (58.3) | 180 (62.9) | 86 (50.6) | **0.010** | 296 (19.4) | 81 (22.7) | 215 (18.4) | 0.072 |
| Rescue and transport of the wounded | 272 (49.7) | 196 (51.6) | 23 (35.4) | 53 (52.0) | **0.048** | 208 (45.6) | 133 (46.5) | 75 (44.1) | 0.621 | 556 (36.4) | 148 (41.5) | 408 (34.9) | **0.024** |
| Epidemic prevention and control | 270 (49.4) | 187 (49.2) | 34 (52.3) | 49 (48.0) | 0.860 | 214 (46.9) | 130 (45.5) | 84 (49.4) | 0.413 | 430 (28.2) | 123 (34.5) | 307 (26.3) | **0.003** |
| Fire disaster | 266 (48.6) | 194 (51.1) | 27 (41.5) | 45 (44.1) | 0.220 | 247 (54.2) | 157 (54.9) | 90 (52.9) | 0.686 | 615 (40.3) | 132 (37.0) | 483 (41.3) | 0.143 |
| Role of field hospital in disaster rescue | 250 (45.7) | 181 (47.6) | 23 (35.4) | 46 (45.1) | 0.185 | 232 (50.9) | 159 (55.6) | 73 (42.9) | **0.009** | 264 (17.3) | 76 (21.3) | 188 (16.1) | **0.023** |
| Flood, typhoon, tsunami, snow disaster, famine, desertification | 248 (45.3) | 185 (48.7) | 25 (38.5) | 38 (37.3) | 0.059 | 243 (53.3) | 149 (52.1) | 94 (55.3) | 0.508 | 458 (30.0) | 124 (34.7) | 334 (28.6) | **0.026** |
| Population vulnerability assessment | 241 (44.1) | 166 (43.7) | 26 (40.0) | 49 (48.0) | 0.574 | 201 (44.1) | 113 (39.5) | 88 (51.8) | **0.011** | 152 (10.0) | 65 (18.2) | 87 (7.4) | **<0.001** |
| Humanitarian responsibility in disaster disposal | 222 (40.6) | 150 (39.5) | 21 (32.3) | 51 (50.0) | 0.055 | 218 (47.8) | 142 (49.7) | 76 (44.7) | 0.307 | 328 (21.5) | 75 (21.0) | 253 (21.6) | 0.799 |
| Mass poisoning | 220 (40.2) | 159 (41.8) | 23 (35.4) | 38 (37.3) | 0.491 | 208 (45.6) | 127 (44.4) | 81 (47.6) | 0.502 | 388 (25.4) | 107 (30.0) | 281 (24.0) | **0.024** |
| Terrorist attacks | 218 (39.9) | 156 (41.1) | 17 (26.2) | 45 (44.1) | **0.048** | 201 (44.1) | 126 (44.1) | 75 (44.1) | 0.990 | 250 (16.4) | 75 (21.0) | 175 (15.0) | **0.007** |
| On-site triage | 207 (37.8) | 153 (40.3) | 22 (33.8) | 32 (31.4) | 0.202 | 177 (38.8) | 112 (39.2) | 65 (38.2) | 0.845 | 353 (23.1) | 99 (27.7) | 254 (21.7) | **0.019** |
| Chemical accidents | 201 (36.7) | 153 (40.3) | 18 (27.7) | 30 (29.4) | **0.036** | 149 (32.7) | 93 (32.5) | 56 (32.9) | 0.926 | 208 (13.6) | 63 (17.6) | 145 (12.4) | **0.011** |
| Mass stampede | 183 (33.5) | 141 (37.1) | 14 (21.5) | 28 (27.5) | **0.018** | 155 (34.0) | 104 (36.4) | 51 (30.0) | 0.165 | 292 (19.1) | 87 (24.4) | 205 (17.5) | **0.004** |
| Traffic accidents | 176 (32.2) | 126 (33.2) | 17 (26.2) | 33 (32.4) | 0.535 | 182 (39.9) | 115 (40.2) | 67 (39.4) | 0.866 | 508 (33.3) | 119 (33.3) | 389 (33.3) | 0.984 |
| Mine disaster | 169 (30.9) | 123 (32.4) | 12 (18.5) | 34 (33.3) | 0.068 | 172 (37.7) | 115 (40.2) | 57 (33.5) | 0.155 | 220 (14.4) | 79 (22.1) | 141 (12.1) | **<0.001** |
| Legal issues in disaster relief | 162 (29.6) | 109 (28.7) | 18 (27.7) | 35 (34.3) | 0.508 | 152 (33.3) | 98 (34.3) | 54 (31.8) | 0.584 | 217 (14.2) | 56 (15.7) | 161 (13.8) | 0.365 |
| Nuclear accidents and radiation accidents | 161 (29.4) | 123 (32.4) | 17 (26.2) | 21 (20.6) | 0.056 | 149 (32.7) | 96 (33.6) | 53 (31.2) | 0.599 | 202 (13.2) | 61 (17.1) | 141 (12.1) | **0.014** |
| Other issues | 109 (19.9) | 82 (21.6) | 11 (16.9) | 16 (15.7) | 0.338 | 115 (25.2) | 79 (27.6) | 36 (21.2) | 0.125 | 180 (11.8) | 47 (13.2) | 133 (11.4) | 0.359 |
