# Supplementary material for: Knowledge Levels and Training Needs of Disaster Medicine among Health Professionals, Medical Students, and Local Residents in Shanghai, China
Source: PLoS One. 2013 Jun 24;8(6):e67041. doi: 10.1371/journal.pone.0067041 (PMC3691157; doi:10.1371/journal.pone.0067041)
Supplement: Questionnaire S1 — Questionnaire for health professionals and medical students. (DOC) [file pone.0067041.s007.doc]

This survey is sponsored by the Second Military Medical University
The questionnaire Information is completely confidential

**-------------------------------------------------------------------------------------------------**

**Disaster Medicine Questionnaire**

**For health professionals and medical students**


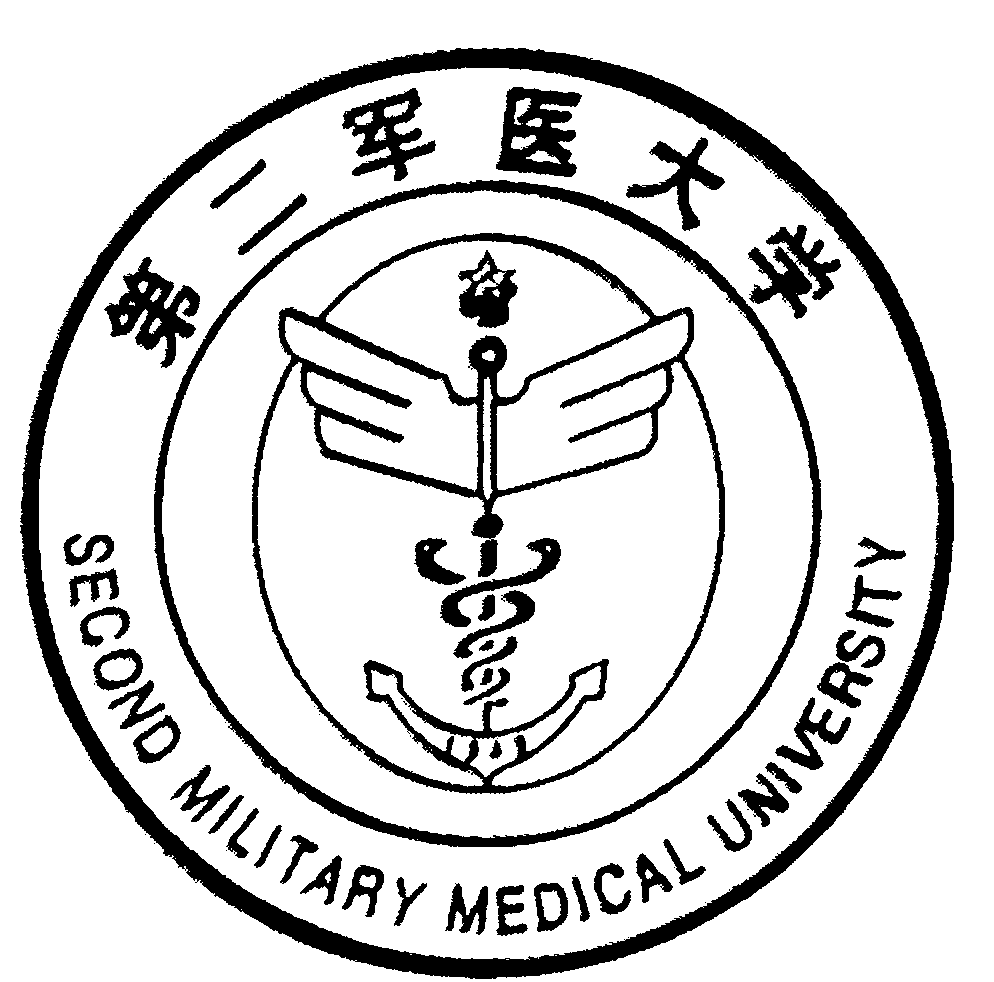


**Number:**

**Supervisor:**

**Date:**

**Disaster is recurrent, especially in crowded areas. Disaster Medicine is a new discipline, the current education system is not perfect. This survey aims to obtain information about the course requirements and improve curriculum design. Your answers will help us to achieve this goal, please answer the questionnaire independently, thank you for your cooperation!**

**Part 1 Personal information**

1. Name:

2. Gender: ①male ②female

3. Date of birth:

4. Age:

5. Educational level: ①junior college ②bachelor ③master ④doctor

6. Telephone:

7. Address:

8. Profession: [1] medical staff: ①clinician ②public health physician ③nurse ④technician

[2] Teacher

[3] Health administrator

9. Professional title : ①junior ②assistant ③associate ④senior；

10. Do you have disaster relief experience? ①yes ②no

11. Have you studied ‘disaster medicine’ course systematically? ①yes ②no

12. What is your self-estimation of disaster medicine knowledge?

① well ② moderate ③ little

13. How do you acquire information about ‘disaster medicine’?

①newspapers / magazines / internet ②TV / radio ③seminar

④school courses ⑤communication with other people

**Part 2 Knowledge**

Multiple-choice questions. Please choose one best answer.

1. On-site treatment is:

A The treatment given at the scene of the accident

B The treatment given in transit

C The treatment given in hospital accident and emergency department

D The treatment given in ICU (intensive care unit)

E The treatment given in patient room

2. Which is the appropriate action for a person trapped in a high-rise fire?

A Yell for help

B Jump out from one window

C Cover mouth and nose with wet towels, crawl along the wall to safety exit

D Hide in the closed office

E Struggle to put out the fire

3. Which of the following procedure is correct during an earthquake?

A Jump out from one window

B Hide in a source of water (eg wash room), waiting for rescue

C Lean against the wall

D Bend over the desk

E Swarm out through the door with the crowd

4. Responsing to a large number of casualties, triage and on-site emergency treatment priority is:

①The wounded has mild traumatic injury, not life-threatening;

②The wounded has life-threatening injury, requiring immediate surgery and having a large chance of survival after treatment;

③The wounded is on the brink of death, all rescue measures are ineffectual.

④The wounded has serious traumatic injury and need treatment, but general condition is stable.

A ③①②④

B ②④①③

C ①④③②

D ②①④③

5. Medical evacuation after an earthquake includes:

A Neighborhood evacuation

B Evacuation over echelon

C Combine military and civilian

D Trans-provincial evacuation

E All above

6. Disaster preparation includes:

A Emergency preplan

B Material reserve

C Community rescue training **and knowledge** propagation

D Population vulnerability assessment

E All above

F None

7. Which of the following post-disaster epidemic prevention strategies is not correct?

A Prevent intestinal infection: wash hands, kill flies, sterilize food and water

B Prevent insect-borne diseases: mosquito control, vaccination

C Prevent respiratory infectious disease: keep warm，use large amounts of antibiotic for prophylaxis

D Prevent post-traumatic diseases: timely injection of tetanus antitoxin，wound debridement, anti-inflammatory

E Prevent zoonosis: rat-proofing, deratization, deinsectization, mosquito control

8. Where should temporary toilets be set in the event of a disaster?

A Upwind, near the source of water

B Downwind, shelter, far from the source of water

C Upwind, conspicuous place

D Downwind, near the source of water, shelter

9. Which of the following is not included in the first aid ABC three steps?

A Keep a patent airway

B Cerebral resuscitation

C Keep effective circulation

D Artificial respiration

10. Which one is wrong about trauma treatment?

A It is necessary for patients in coma to keep airway open.

B Open fracture reduction should be done on the spot.

C Patients with artery ruptures should be bandaged to stop bleeding.

D Patients with spinal fractures should be kept straight.

11. The first step of cardiopulmonary resuscitation is:

A Supply oxygen

B Closed cardiac massage

C Artificial respiration

D Open airway

E Cerebral resuscitation

12. Which one is wrong about hemostasis with tourniquet?

A Hemostasis with tourniquet applies to bleeding limbs.

B Tourniquet should be directly tied at limbs.

C The tightness of tourniquet is appropriate when distal pulse can not be touched.

D The ligature time is not more than 1 hour or 1~2 minutes’ slack every 1 hour ligature.

E It is necessary for transported patients to have clear signs to make sure ligature time.

13. Which question psychological aid-givers shouldn't ask survivors immediately after the disaster:

A I feel very sad for your experience of pain and danger.

B You're safe now (if the person is really safe).

C It is not your fault. It is normal that you feel this way.

D Restrain your emotion, it is very lucky to survive.

E Now you can express all your feelings, you can cry or anger.

14. Which of the following is not diagnostic basis of post-traumatic stress disorder (PTSD)?

A Recall or dream of traumatic events repeatedly and painfully

B Try to avoid the concerned trauma feelings or talk about them

C Rarely participate in meaningful activities or lose interest in social activities

D Symptoms develop within one week after traumatic events

E Difficulty falling asleep, irritability, difficulty of concentration

15. The biggest difference between disaster relief in remote areas and urban rescue is:

A Rating evacuation

B Rural rescue team should include various types of professionals and adequate supplies

C Evacuation by helicopter

D Maintain smooth communications and transportation

E Need cooperation of combat troops

16. Which one is not included in population vulnerability assessment?

A Popularize disaster rescue knowledge in community

B People’s self-aid capacity in disaster rescue

C The improvement of laws and regulations

D Emergency reservation and preplan

E The extent and type of disaster

F None

**Part 3 Demand**

Multiple-choice questions. Please choose one or more answer.

1. How do you want to learn disaster medicine?

A Systemic study (classroom study)

B Lecture

C Academic report

D Practical training

E Watch disaster movies or videos

2. Which of the following do you think disaster medicine should be included in?

A Required course for clinician

B Required course for public health professional

C Selective course for clinician

D Selective course for public health professional

E Informal course

3. Which kind of teaching materials for disaster medicine course do you want to use?

A Handouts for internal use

B National unified textbook

C Foreign teaching materials

D Military teaching materials

E Other

4. What do you think is (are) more important in disaster medicine learning?

A Fundamental principle of disaster medicine

B Principle of disaster disposal

C First aid skills

D Triage, evacuation, and referral

E Post-disaster epidemic prevention

F Post-disaster psychological disorder

G Disaster medical supervision

5. The contents that you are interested in and want to further learn are:

(1) National and local disaster reduction plans and preparedness against disaster

(2) Humanitarian responsibility in disaster disposal

(3) Basic principles of disaster assistance

(4) The role of modern information technology in disaster assistance

(5) The role of field hospitals in disaster assistance

(6) Rear support hospitals' tasks and preparation in disaster assistance

(7) Treatment principles and first-aid skills

(8) The rescue and transport of the wounded

(9) On-site triage

(10) Traffic accidents

(11) Earthquakes

(12) Mine disaster

(13) Terrorist attacks

(14) Mass poisoning

(15) Flood disaster, typhoon, tsunami, snow damage, famine, desertification

(16) Groups stampede

(17) Subway and tunnel emergency accident

(18) Fire disaster

(19) Chemical Accidents

(20) Nuclear accidents and radiation Accidents

(21) Legal issues of disaster assistance

(22) Medical issues in disaster phase

(23) Population vulnerability assessment

(24) Post-disaster psychological relief

(25) Post-disaster epidemic prevention
