# Supplementary material for: Knowledge Levels and Training Needs of Disaster Medicine among Health Professionals, Medical Students, and Local Residents in Shanghai, China
Source: PLoS One. 2013 Jun 24;8(6):e67041. doi: 10.1371/journal.pone.0067041 (PMC3691157; doi:10.1371/journal.pone.0067041)
Supplement: Questionnaire S2 — Questionnaire for community residents. (DOC) [file pone.0067041.s008.doc]

This survey is sponsored by the Second Military Medical University
The questionnaire Information is completely confidential

**-------------------------------------------------------------------------------------------------**

**Disaster Medicine Questionnaire**

**For community residents**


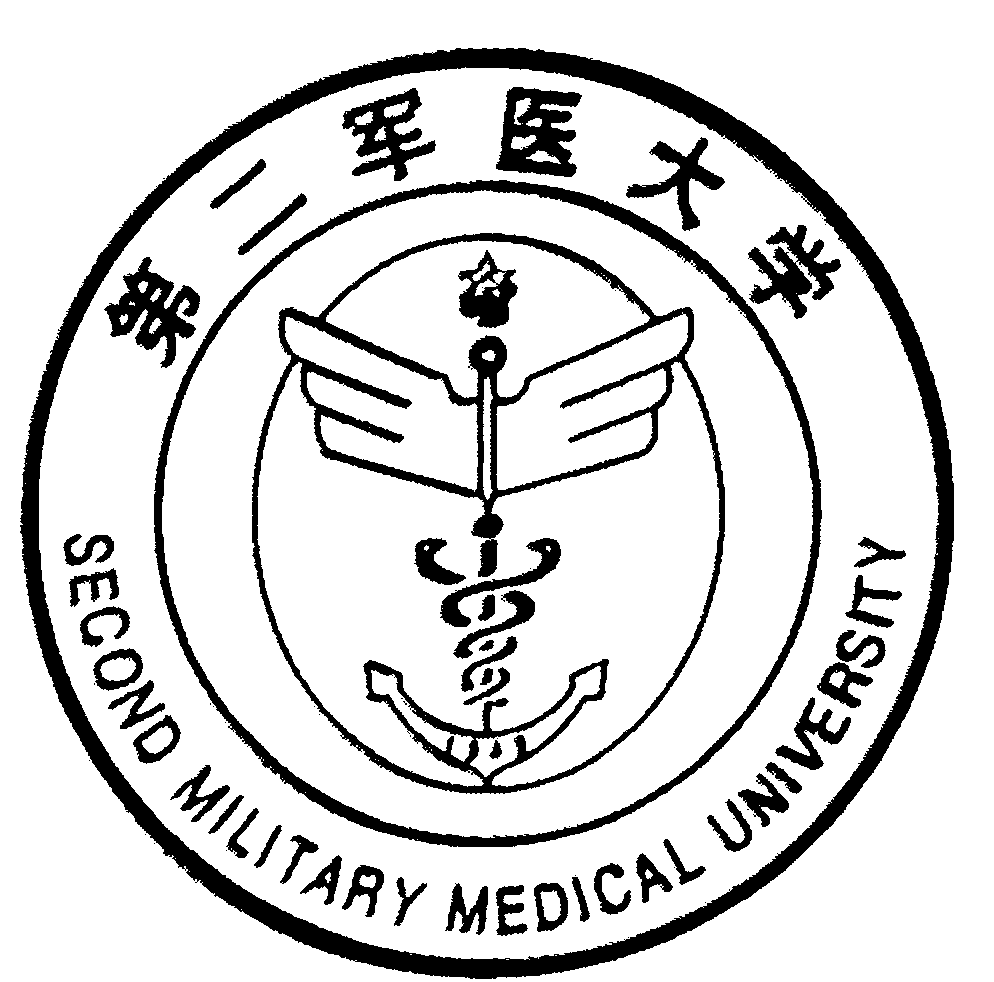


**Number:**

**Supervisor:**

**Date:**

**Disaster is recurrent, especially in crowded areas. Disaster Medicine is a new discipline, the current education system is not perfect. This survey aims to obtain information about the course requirements and improve curriculum design. Your answers will help us to achieve this goal, please answer the questionnaire independently, thank you for your cooperation!**

**Part 1 Personal information**

1. Gender: ①male ②female

2. Age:

3. Educational level: ①junior college ②bachelor ③master or higher ④others

4. Profession: ①employee ②civil servant ③teacher ④others

5. Do you have disaster relief experience? ①yes ②no

6. What is your self-estimation of disaster medicine knowledge?

①well ②moderate ③little

7. How do you acquire information about ‘disaster medicine’?

①newspapers / magazines / internet ②TV / radio ③seminar

④school education ⑤communication with other people

**Part 2 Choice questions**

Multiple-choice questions. Please choose one best answer.

1. Which set of emergency call numbers is all correct?

A 119--emergency call; 110--fire alarm; 120--first-aid center; 122--traffic accident

B 110--emergency call; 119--fire alarm; 120--first-aid center; 122--traffic accident

C 120--emergency call; 119--fire alarm; 110--first-aid center; 122--traffic accident

D 110--emergency call; 119--fire alarm; 122--first-aid center; 120--traffic accident

2. Will you initiatively pay attention to the position of exit passageway in public places?

A Yes B No C It does not matter

3. Which one is correct about self-rescue measures in a nuclear leak?

A Conceal; Rapid evacuation; Pay attention to food and water; Necessary medication protection.

B Conceal; Rapid evacuation; Does not need to consider food, water and medication protection.

C Does not need to conceal, rapid evacuation and consider medication protection; Pay attention to food and water.

D Does not need to conceal and protect.

4. The first step of cardiopulmonary resuscitation is:

A Supply oxygen

B Closed cardiac massage

C Artificial respiration

D Open airway

E Cerebral resuscitation

5. The main difference between disaster relief in remote areas and urban rescue is:

A Rating evacuation

B Rural rescue team should include various types of professionals and adequate supplies

C Evacuation by helicopter

D Maintain smooth communications and transportation

E Need cooperation of combat troops

6. Which one is not correct about trauma treatment?

A It is necessary for patients in coma to keep airway open.

B Open fracture reduction should be done on the spot.

C Patients with artery ruptures should be bandaged to stop bleeding.

D Patients with spinal fractures should be kept straight.

7. Which one is right for a person trapped in a high-rise fire?

A Yell for help

B Jump out from one window

C Cover mouth and nose with wet towels, crawl along the wall to safety exit

D Hide in the closed office

E Struggle to put out the fire

8. Which of the following procedure is correct during an earthquake?

A Jump out from one window

B Hide in a source of water (eg wash room), waiting for rescue

C Lean against the wall

D Bend over the desk

E Swarm out through the door with the crowd

9. Where should temporary toilets be set in the event of a disaster?

A Upwind, near the source of water

B Downwind, shelter, far from the source of water

C Upwind, conspicuous place

D Downwind, near the source of water, shelter

10. Which question psychological aid-givers shouldn't ask survivors immediately after the disaster:

A I feel very sad for your experience of pain and danger.

B You're safe now (if the person is really safe).

C It is not your fault. It is normal that you have such a feeling.

D Restrain your emotion, it is very lucky to survive.

E Now you can express all your feelings, you can cry or anger.

11. Which of the following post-disaster epidemic prevention strategies is not correct?

A Prevent intestinal infection: wash hands, kill flies, sterilize food and water

B Prevent insect-borne diseases: mosquito control, vaccination

C Prevent respiratory infectious disease: keep warm，use large amounts of antibiotic for prophylaxis

D Prevent post-traumatic diseases: timely injection of tetanus antitoxin，wound debridement, anti-inflammatory

**Part 3 Demand survey**

Multiple-choice questions. Please choose one or more answer.

12. Do you think it is necessary to learn disaster medicine?

A Yes B No C It does not matter

13. How do you want to learn disaster medicine?

A Systemic study (classroom study)

B Lecture

C Academic report

D Practical training

E Watch disaster movies or videos

14. Do you think it is necessary to develop disaster medicine course for children?

A Yes B No C It does not matter

15. Would you like to participate in disaster simulation drills organized in community?

A I am willing to participate regularly.

B I am willing to participate occasionally.

C I am not willing to participate.

D It does not matter.

16. Do you think it is necessary to set up community volunteer team for disaster relief? And would you like to become one of the volunteers?

A It should be set up, and I am willing to participate.

B It should be set up, but I am not willing to participate.

C It should not be set up, and I am not willing to participate.

D It does not matter.

17. What do you think is (are) more important in disaster medicine learning?

A Fundamental principle of disaster medicine

B Principle of disaster disposal

C First aid skills

D Triage, evacuation, and referral

E Post-disaster epidemic prevention

F Post-disaster psychological disorder

G Disaster medical supervision

18. The contents that you are interested in and want to learn more are:

(1) National and local disaster reduction plans and preparedness against disaster

(2) Humanitarian responsibility in disaster disposal

(3) Basic principles of disaster assistance

(4) Role of modern information technology in disaster assistance

(5) Role of field hospitals in disaster assistance

(6) Rear support hospitals' tasks and preparation in disaster assistance

(7) Treatment principles and first-aid skills

(8) The rescue and transport of the wounded

(9) On-site triage

(10) Traffic accidents

(11) Earthquakes

(12) Mine disaster

(13) Terrorist attacks

(14) Mass poisoning

(15) Flood disaster, typhoon, tsunami, snow damage, famine, desertification

(16) Groups stampede

(17) Subway and tunnel emergency accident

(18) Fire disaster

(19) Chemical Accidents

(20) Nuclear accidents and radiation Accidents

(21) Legal issues of disaster assistance

(22) Medical issues in disaster phase

(23) Population vulnerability assessment

(24) Post-disaster psychological relief

(25) Post-disaster epidemic prevention
